# Carbonate apatite nanoparticles carry siRNA(s) targeting growth factor receptor genes *egfr1* and *erbb2* to regress mouse breast tumor

**DOI:** 10.1080/10717544.2017.1396385

**Published:** 2017-11-09

**Authors:** Snigdha Tiash, Nur Izyani Binti Kamaruzman, Ezharul Hoque Chowdhury

**Affiliations:** Jeffrey Cheah School of Medicine and Health Sciences, Monash University Malaysia, Jalan Lagoon Selatan, Bandar Sunway, Malaysia

**Keywords:** Growth factor receptors, siRNA, carbonate apatite nanoparticles (NPs), gene silencing, breast cancer, tumor regression

## Abstract

Cancer cells lose their control on cell cycle by numerous genetic and epigenetic alterations. In a tumor, these cells highly express growth factor receptors (GFRs), eliciting growth, and cell division. Among the GFRs, epidermal growth factor receptor-1 (EGFR1) (Her1/ERBB1) and epidermal growth factor receptor-2 (EGFR2) (Her2/ERBB2) from epidermal growth factor (EGF) family and insulin-like growth factor-1 receptor (IGF1R) are highly expressed on breast cancer cells, thus contributing to the aggressive growth and invasiveness, have been focused in this study. Moreover, overexpression of these receptors is related to suppression of cell death and conferring resistance against the classical drugs used to treat cancer nowadays. Therefore, silencing of these GFRs-encoding genes by using selective small interfering RNAs (siRNAs) could be a powerful approach to treat breast cancer. The inorganic pH sensitive carbonate apatite nanoparticles (NPs) were used as a nano-carrier to deliver siRNA(s) against single or multiple *GFR* genes in breast cancer cells as well as in a mouse model of breast carcinoma. Silencing of *egfr1* and *erbb2* simultaneously led to a reduction in cell viability with an increase in cell death signal in the cancer cells and regression of tumor growth *in vivo*.

## Introduction

Cancer, medically known as malignant neoplasia, is a broad group of diseases involving unregulated cell growth as a characteristic signature for cancer development and progression. Growth signal is initiated in cells upon binding of growth factors to GFRs expressed on cell surface, accelerating cell growth, division, and proliferation (Waterfield, 1989). On cancer cells, there is very high expression of these receptors, thus enhancing the signal and leading to unrestricted cell growth. In this study, we have paid attention to three different GFRs that are highly expressed on breast cancer, namely epidermal growth factor receptors (EGFR1 and ERBB2) and insulin-like growth factor 1 receptor (IGF1R).

EGFR1 (ERBB1/Her1) is the cell surface protein of the members of epidermal growth factor (EGF) family. Overexpression of wild type EGFR1 is observed in breast cancer (14–91%) (Klijn et al., 1992; Allen et al., 2002; Herbst and Shin, 2002). The 170-KDa single polypeptide chain of EGFR1 encoded by *egfr1* gene consists of three domains, with the extracellular and intracellular domains separated by a transmembrane domain. Upon binding with the ligands, the receptors form homodimers or heterodimers with other receptors of EGF family and relay the signal inside cells to trigger the tyrosine kinase activity to phosphorylate and activate a number of downstream proteins that play important roles in cell division and proliferation (Voldborg et al., 1997). Overexpression of EGFR1 protein in cancer cells occurs through gene duplication or mutation via genetic rearrangement (Nahta et al., 2003). In addition, expression of constitutively active truncated EGFR vII protein that lacks extracellular domain in breast cancer (20–78%) contributes to the aggressiveness of tumor (Voldborg et al., 1997; Allen et al., 2002).

Encoded by *erbb2* gene, the second member of EGF family known as ERBB2/Her2 (also EGFR2) is a transmembrane glycoprotein having very similar structure of EGFR1. Upon binding with ligands, it forms heterodimers with EGFR1 and relays growth signal in the cells. ERBB2 is, however, unable to form homodimers. Overexpression of ERBB2 through gene amplification is noticed in breast cancer (30%) and even in other malignancies (Slamon et al., 1987), correlating with poor disease prognosis (Guy et al., 1996). Although no mutation in *erbb2* gene has been identified in cancer (Nahta et al., 2003), ERBB2 acts as a potent oncoprotein both *in vitro* and *in vivo* (Di Fiore et al., 1987; Guy et al., 1992; Guy et al., 1996), with the mechanism for gene amplification yet to be revealed.

IGF1R binds with insulin-like growth factor 1 (IGF1) with higher affinity than insulin-like growth factor 2 (IGF2). Encoded by *igf1r* gene, a 180-KDa precursor protein is synthesized, post-translationally modified to a polypeptide containing one α-chain and one β-chain connected by disulfide bonds, and expressed on cell surface. The α-chain and portion of the β-chain comprise the extracellular domain followed by transmembrane and cytoplasmic domain in the β-chain. The mature IGF1R is a homodimer comprising α_2_β_2_ chain of 320-KDa linked by disulfide bond. The intracellular domain contains the tyrosine kinase activity that autophosphorylates the protein and several downstream proteins to relay signal after binding to its ligands. Overexpression of *igf1r* gene is implicated in cellular proliferation, transformation, and metastasis in several carcinomas, including breast cancer (Yee, 2002; Nahta et al., 2003).

These GFRs not only contribute to the aggressive cell growth in tumor, but also confer resistance to classical drugs by different mechanisms and cross-talks in the signaling pathways. For example, up-regulation of erbb2 expression in presence of hormonal therapy (e.g. tamoxifen) leads to tamoxifen-resistant breast cancer (Knowlden et al., 2003; Gee et al., 2005; Hurtado et al., 2008; Yonesaka et al., 2011). The relevance of GFRs and their functions in breast cancer are well established. Interfering the signaling pathways initiated by these receptors with different monoclonal antibodies or inhibitors in several ongoing clinical trials shows development of resistance to chemotherapy with possible side effects. Moreover, cross-talks between the different GFRs-mediated signals could account for acquired resistance against a drug that targets one particular GFR. Thus, enhancement of ERBB2-mediated signal in EGFR1-directed antibody (cetuximab) and IGF1R-mediated signal in anti-erbB2/HER2 (trastuzumab) antibody-treated breast cancer patients confers resistance to the treatment (Nahta et al., 2005; Yonesaka et al., 2011). Therefore, targeted silencing of the single or multiple genes encoding GFRs would be an attractive therapeutic approach in combating breast cancer.

Selective silencing of these *GFR* genes by using exogenous synthetic small interfering RNAs (siRNAs) to suppress growth of cancer cells demands a suitable carrier to deliver the anionic siRNA through the negatively charged cell membrane. The pH sensitive carbonate apatite nanoparticles (NPs) having strong affinity towards anionic nucleic acids (DNA or siRNA) with excellent biocompatibility, favorable pharmacokinetics, high tumor accumulation capacity and rapid, intracellular release rate through proton-driven self-dissolution, recently emerged as an attractive nano-career to efficiently carry siRNAs in diverse cancer cell lines and animal models to silence particular endogenous genes (Hossain et al., 2010; Chua et al., 2013; Kunnath et al., 2014a,b), and, therefore, have been employed here to carry the single or multiple siRNA(s) in breast cancer cells as well as in breast tumors subcutaneously induced in mice mammary pads. It has been revealed that silencing of *GFR* genes reduced cell viability (CV), with the highest cytotoxicity observed with the siRNAs targeting *egfr1* and *erbb2* genes, enhanced apoptotic signal in the cells and also reduced the tumor volume in mice, with exception of *igf1r* gene knockdown. Thus, this study suggests that silencing of *egfr1* and *erbb2* genes through delivery of their target siRNAs with carbonate apatite NPs is a promising therapeutic modality to treat breast cancer.

## Materials and methods

### Materials

Dulbecco’s modified eagle medium (DMEM), calcium chloride dehydrate (CaCl_2_.2H_2_O), sodium bicarbonate (NaHCO_3_), dimethyl sulphoxide (DMSO), thiazolyl blue tetrazolium bromide (MTT), and ethylene diamine tetraacetic acid (EDTA) were purchased from Sigma-Aldrich (St. Louis, MO). DMEM powder, fetal bovine serum (FBS), trypsin-ethylenediamine tetraacetate (trypsin-EDTA), and penicillin-streptomycin were obtained from Gibco BRL (CA). Caspase-glo 3/7 kit was purchased from Promega (Madison, WI). All siRNAs that have been used in this study and listed in Table 1 were obtained from Qiagen and dissolved in RNase-free water provided by the company to obtain 10 µM stock solution. MCF-7 and 4T1 cells were originally from ATCC.

**Table 1.** siRNAs used in this study.

Name	Target sequence	Targeted gene	Validation cell line	% Knock down
Allstars negative control siRNA	Proprietary	Proprietary	N/A	–
Allstars negative siRNA AF 488 (3′)	Proprietary	Proprietary	N/A	–
Hs_EGFR_10	TACGAATATTAAACACTTCAA	*egfr1*	HeLa	80
Hs_ERBB2_14	AACAAAGAAATCTTAGACGAA	*erbb2*	MCF7	92
Hs_IGF1R_1	ATGGAGAATAATCCAGTCCTA	*igf1r*	N/A	N/A

### Preparation of carbonate apatite NPs-bound siRNA(s)

For *in vitro* study, NPs were prepared as described earlier (Tiash et al., 2014). Briefly, NPs were formed in 1 mL of freshly prepared bicarbonated (44 mM) DMEM media (pH 7.4) by mixing 3 mM of exogenous CaCl_2_ (3 µL from 1 M stock), followed by incubation of the mixture for 30 min at 37 °C. For making complexes with siRNA(s) (NPs-siRNA(s)), 1 nM of either allstars negative control siRNA or functionally validated siRNA(s) against single or multiple *GFR* genes were added to 1 mL of DMEM media along with 3 mM of CaCl_2_ prior to 30 min incubation at 37 °C. For making NPs-complexes of fluorescent negative siRNA, 5 nM or 10 nM of allstars negative control siRNA AF488 (3′) was used for the studies of siRNA binding affinity for NPs and cellular uptake. After 30 min of incubation at 37 °C, 10% of FBS was added to all samples to prevent aggregation.

For animal study, NPs were prepared in 200 µL of freshly prepared DMEM media mixed with 14 µL of 1 M CaCl_2_ (70 mM of Ca^2+^) in small tubes and incubated for 30 min at 37 °C. As many as 50 nM of each siRNA against all three *GFR* genes or negative control siRNA was used to prepare complexes in 200 mL DMEM media through incubation at 37 °C for 30 min. After preparation of NPs and NPs-siRNA(s), all samples were maintained on ice till the injection period to prevent aggregation.

### Characterization of NPs by Fourier transform infrared spectroscopy (FT-IR) and X-ray diffraction (XRD)

Carbonate apatite NPs were generated and precipitated in 1 L of DMEM media supplemented with sodium bicarbonate and 40 mM of CaCl_2_. The precipitates were collected in 50 mL centrifuge tubes and subjected to centrifugation at 1035 rpm by using Allegra X-12 centrifuge (Beckman Coulter, Fullerton, CA). The supernatant was discarded and milli-Q water was added to wash the precipitates before repeating the centrifugation process. This step was repeated for several times and the sample was sent for lyophilization. The powder thereby produced was analyzed via FT-IR and XRD (inXitu, Mountain View, CA).

### Acid dissolution of carbonate apatite NPs

NPs were formulated at pH 7.5 with 8 mM of CaCl_2_ in bicarbonate-buffered media by incubating for 30 min at 37 °C. After the particles were formed, the absorbance was taken by using the spectrophotometer at 320 nm. As many as 1 N HCl was used to reduce the pH of the solution containing the particles. Data were represented as mean ± SE for triplicates.

### Measurement of particle size

Size of the NPs formed in different concentrations of CaCl_2_ was measured using Zeta Sizer (Nano ZS, Malvern, Worcestershire, UK) after adding 10% of FBS and temporarily keeping on ice to prevent particle aggregation. A refractive index ratio of 1.325 was set for the estimation of particle diameter. Data were analyzed using Zetasizer software 6.20 (Worcestershire, UK) and all samples were measured in duplicate.

### Assessment of binding affinity of siRNA to NPs

AF 488 allstars negative siRNAs at different concentrations (nM scale) were dissolved in bicarbonated DMEM media. Fluorescence intensity was measured for 100 µL of solution in 2030 multilabel reader vitorTM X5 (Perkin Elmer, MA, USA) attached with Perkin Elmer 2030 manager software using λex =490 nm and λem =535 nm. Samples were blank corrected using 100 µL of DMEM media only. Each experiment was completed in duplicate and repeated two times to check reproducibility. Standard curve was obtained by fluorescence intensity versus concentrations of siRNA used (supplementary Figure 3). The correlation coefficient for this graph is 0.98. The curve was used to calculate the concentrations of siRNA present in the supernatant of the samples used in successive study. For making NPs-siRNA, 5 nM of AF 488 negative siRNA was added to the DMEM prior to the addition of 1–7 mM of exogenous CaCl_2_, followed by incubation at 37 °C for 30 min. Subsequently, the samples were centrifuged at 13,200 rpm for 30 min at 4 °C. One hundred microliter of the collected supernatant was subjected to fluorescence intensity measurement. The concentration of siRNA present in the supernatant was calculated using the standard curve and the binding affinity of siRNA for NPs was calculated using the following formula:

$$\text{\%}\;\text{Interaction}\;\text{efficiency}=\frac{{\left[X\right]}_{\text{free}\;\text{siRNA}}-{\left[X\right]}_{\text{NPs-siRNA}}}{{\left[X\right]}_{\text{initial}}}\;\times 100,$$

where [X]_free siRNA_ and [X]_NPs-siRNA_ are the concentrations of siRNA in the supernatant following centrifugation of free and NPs-siRNA, respectively (calculated from the standard curves), and [X]_initial_ is the total concentration of siRNA used for the experiment, which was 5 nM. Each experiment was completed in triplicate and shown as mean ± SD.

### Observation of cellular uptake of NPs-carried siRNA

For observing the cellular uptake, cells were treated for 6 h with media alone (untreated), NPs formed with 3 mM of CaCl_2_, free siRNA (10 nM fluorescent negative siRNA), and NPs-siRNA formed with 10 nM fluorescent negative siRNA and 3 mM of CaCl_2_. After the treatment, cells were washed with 5 mM EDTA in PBS to remove extracellular NPs and NPs-siRNA and observed under fluorescent microscope (Olympus DP73, Tokyo, Japan).

Additionally, treated cells were washed with 5 mM EDTA in PBS and lysed before fluorescence intensity was measured for the lysate in 2030 multilabel reader vitorTM X5 (Perkin Elmer) attached with Perkin Elmer 2030 manager software using λex = 490 nm and λem = 535 nm. Samples were blank corrected using untreated samples. The experiment was completed in duplicate and expressed as mean ± SD. Statistical analysis was completed among different treatment groups.

### CV assay by 3-(4,5-dimethylthiazol-2-yl)-2,5-diphenyltetrazolium bromide (MTT)

Human breast cancer cell line, MCF-7, was cultured in a 75 cm^2^ tissue culture flasks (Nunc, Orlando, FL) and maintained in the DMEM media (pH 7.4) supplemented with 10% of FBS, and penicillin and streptomycin antibiotics, in a CO_2_ incubator. Approximately 5 × 10^4^ cells were seeded on 24-well plates (Greiner, Frickenhausen, Germany). After 24 h, the cells were treated with media (untreated), NPs formed with 3 mM of CaCl_2_, and NPs-siRNA(s) formed using 3 mM of CaCl_2_ and 1 nM of each of single siRNA or multiple siRNAs, for two consecutive days. Lastly, 50 µL of MTT (5 mg/mL in PBS) was added to each well so that metabolically active cells could form formazan crystals which were subsequently (after 4 h) dissolved by adding 300 µL of DMSO. The plates were then agitated on built-in plate shaker for 20 s. Optical density (OD) at 595 nm wavelength with 630 nm of reference wavelength was measured using a multiwell plate reader (microplate spectrophotometer, Biorad, Hercules, CA) for formazan quantification, and % of metabolically active cells (CV) was calculated for treated samples using the following equation:

$$\text{\%}\;\text{of}\;\text{cell}\;\text{viability}\;\text{(CV)}=\frac{{OD}_{\text{treated}}-{OD}_{\text{reference}}}{{OD}_{\text{untreated}}-{OD}_{\text{reference}}}\;\times 100.$$

Each experiment was completed in triplicate and expressed in graphs as mean ± SD of % of CV. Statistical analysis was completed between NPs treatment and different single or multiple NPs-bound siRNA(s) groups.

All the experiments were repeated for three times and cytotoxicity enhancement (%) due to siRNA(s) entrapment in NPs was calculated using the following formula for all the experiments and expressed as mean ± SD.

$$\text{\%}\;\text{Enhancement}\;\text{in}\;\text{cytotoxicity}\;\text{(\%)}={CV}_{\text{NPs}}-{CV}_{\text{NPs-siRNA}}.$$

### Caspase assay

MCF-7 cells from exponentially growth phase were seeded in 96-well white walled optical bottom plates (Nunc, Germany) containing 1 × 10^4^ cells in each well. After 24 h, cells were treated with 200 µL of media (untreated), NPs formed with 3 mM of CaCl_2_ and NPs complexes of siRNA against single *GFR* gene formed with 3 mM of CaCl_2_ and 1 nM of either of three GFR siRNAs or negative control siRNA in DMEM media. After 2-d treatment, 100 μL of pro-luminescent Caspase-Glo 3/7 substrate which contains the tetrapeptide sequence DEVD (Aspartic acid-Glutamic acid-Valine- Aspartic acid) was added in an ‘add-mix-measure’ format to the cells and mixed by pipetting, resulting in cell lysis and release of ‘glow-type’ luminescent signal via cleavage of the substrate mediated by the caspase enzymes (caspases 3 and 7) available in the cells and cell milieu. After 3-h incubation of the cells in presence of the substrate at 37 °C, luminescence signal was counted by 2030 multilabel reader vitor™ X5 (Perkin Elmer) attached with Perkin Elmer 2030 manager software. As the relative light per unit (RLU) was directly correlated with the amount of the caspases present in the sample, data were represented as mean ± SD of RLU of the luminescence for duplicate samples. Statistical analysis was performed between NPs treatment and different NPs-bound siRNA groups.

### 4T1-induced breast cancer murine model and treatment

Female Balb/c mice (6–8 weeks old) of 15–20 g of body weights were obtained from School of Medicine and Health Science Animal Facility, Monash University. The mice were maintained in 12:12 light:dark condition and provided with ad libitum and water. All the experiments were completed in accordance with the guidelines set by Monash University Animal Welfare Committee. Mouse breast cancer cell line, 4T1, was maintained in DMEM media supplemented with 10% of FBS and 1% of penicillin and streptomycin antibiotics. For induction of breast tumor, approximately 1 × 10^5^ cells (in 100 µL PBS) were injected subcutaneously on the mammary pad of mice (considered as day 1) and the mice were checked daily for the palpable presence of tumor by index finger. Mice were grouped (six mice per group) randomly when the tumor outgrowth reached to an average volume of 13.20 ± 2.51 mm^3^, and treated intravenously (tail-vein) in the right or left caudal vein. The second dose was administered after 3 d of the first dose.

**Table 2.** Enhancement of cytotoxicity (%) due to complexation using 1 nM of siRNA(s) against *GFR* genes with NPs in MCF-7 cells. The enhancement values for NPs-siRNA(s) were compared to NPs (to confirm the effect of siRNA entrapment into complex structure).

siRNA against	% of cytotoxicity enhancement
*egfr1*	12.03 ± 1.8
*erbb2*	5.6 ± 0.5
*igf1r*	5.47 ± 2.8
*egfr1*; *erbb2*	25.67 ± 4.72
*egfr1*; *igf1r*	4.48 ± 3.5
*erbb2*; *igf1r*	0
*egfr1*; *erbb2*; *igf1r*	7.49 ± 4.9

%  in cytotoxicity enhancement =CV_NPs_-CV_NPs-siRNA_. Data were represented as mean ± SD for triplicate samples. ‘0’ denotes ‘no effect’.

The lengths and widths of the outgrowth tumor were measured using the vernier caliper in millimeter scale over a period of 30 d at regular interval and the volume of the tumor was calculated using the following formula (Kunnath et al., 2014a,b):

Tumor volume (mm3) = 1/2 (length × width2).

The data have been presented here as mean ± SD of tumor volumes of six different mice from each group. Statistical analysis was completed among different treatment groups.

The gross body weights of mice were also monitored and scrutinized with regard to their daily activities and then humanly sacrificed by cervical dislocation at the end of study.

### Statistical analysis

Statistical analysis was completed using the SPSS (version 17 for Windows, North Castle, NY). Two-sample *t*-test was performed for *in vitro* data and LSD *post hoc* test for one-way ANOVA was used for *in vivo* data to analyze and compare the significant difference. Data were considered statistically significant when * or #*p* < .05 and **p < .001.

## Results and discussion

### Characterization of carbonate apatite NPs by FT-IR and XRD

The formation of CA was confirmed via FT-IR where it involves the vibration of molecules targeted via the infrared spectroscopy on the lyophilized CA sample. The broad adsorption between 3343 and 3333 cm^−1^ and 1657 and 1644 cm^−1^ are indications of adsorbed water (supplementary Figure 1a). The spectrum is also showing peaks that represent the carbonate (1416 and 868 cm^−1^) and phosphate (1032, 585, and 561 cm^−1^) in the poorly crystalline apatite, which are in agreement with the XRD result. Less crystalline apatite is the main mineral component of mineralized tissues in vertebrates such as in bones, teeth, soft tissues, and cartilages, thus the generated CA may not be foreign in the physiological environment of the organisms. XRD analysis was also completed to evaluate the apatite features on the CA sample prepared. Supplementary Figure 1(b) shows broad diffraction peaks representing regular pattern of a poorly crystalline apatite that was observed for the CA powder sample.

**Figure 1.**
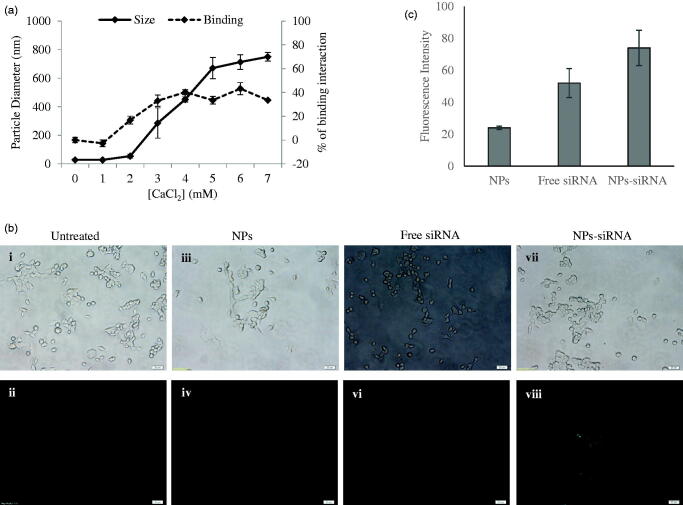
(a) Size of NPs and binding interaction of negative siRNA with NPs formed with different conc. of CaCl_2_. NPs were formed with 1–7 mM of exogenous CaCl_2_ and 5 nM of AF488 negative control siRNA. (b) Cellular uptake of NPs-bound fluorescent negative siRNA. MCF-7 cells were treated with (i, ii) media (untreated), (iii, iv) NPs, (v, vi) free siRNA, and (vii, viii) NPs-siRNA formed with 3 mM of CaCl_2_ and 10 nM AF488 negative control siRNA. Photos were captured at 40× magnification. Upper panel-light images, bottom panel-fluorescent images. Bar indicates 20 µm scale. (c) Fluorescence intensity of intracellular components. MCF-7 cells were treated with NPs, free siRNA, and NPs-siRNA formed with 3 mM of CaCl_2_ and 10 nM AF488 negative control siRNA. After 6 h, fluorescence intensity of cell lysates was measured. Values are represented for duplicate samples: *p < .05 compared to NPs treatment.

### siRNA efficiently interacts with carbonate apatite NPs

It is always imperative to check binding affinity of NPs toward siRNA to confirm the experimental design with sufficient loading capacity. As many as 5 nM of fluorescent negative siRNA was used to check the difference of interactions between the siRNA and the NPs formed with increasing concentrations of CaCl_2_ (Figure 1(a): right *y*-axis, dotted line) with other reactants for particle formation (i.e. inorganic phosphate and bicarbonate) kept constant. The interaction efficiency of siRNA with NPs increased from 2 mM of Ca^2+^ and reached to a plateau level at 4 mM of Ca^2+^. Particle diameter was also measured and found to increase sharply for Ca^2+^ used from 2 mM to 5 mM and continue to increase steadily up to 7 mM (Figure 1(a): left *y*-axis, solid line).

Negative charges on the phosphate backbone of siRNA could bind very efficiently to the cationic Ca^2+^-rich domain of the carbonate apatite NPs through ionic interactions (Hossain et al., 2010). With increasing concentration of Ca^2+^ to 4 mM, siRNA binding reached to plateau level, indicating that the binding of siRNA to NPs became saturated under the fixed siRNA concentration, while the particle size was still increasing with a rise in Ca^2+^ concentration. The particle size increased by aggregation with higher concentration of CaCl_2_, while depletion of inorganic phosphate which is present at 0.9 mM in the medium probably resulted in slow growth of the particles without a further increase in average particle diameter. These aggregated particles might lead to limited siRNA binding as the surface area of the particles decreases with aggregation.

### Acid dissolution pattern of carbonate apatite NPs

A prerequisite to siRNA-mediated knockdown of a target mRNA is efficient release of siRNA payload from the carrier. In order to verify whether carbonate apatite, NPs are sensitive to degradation at acidic pH of endosomes in order for ensuring fast release of the electrostatically associated siRNA, we have carried out pH-dependent dissolution of the particles. As shown in Supplementary Figure 2, with a decrease in pH, there was a sharp decrease in turbidity as a reflection of dissolution of the pre-formed particles, suggesting that siRNA could be released from the particles after being internalized by target cells via endocytosis.

**Figure 2.**
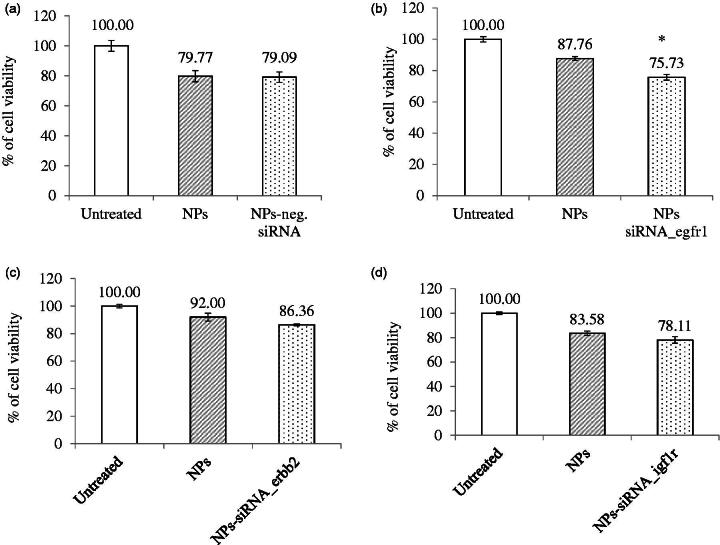
Effect of NPs-loaded siRNA against single gene on viability of MCF-7 cells. Cells were treated with media (untreated), NPs, and NPs-siRNA (1 nM) formed with 3 mM CaCl_2_. Values are represented as % of cell viability compared to untreated cells for triplicate samples. (a) NPs-negative siRNA and siRNA against; (b) *egfr1*; (c) *erbb2*; and (d) *igf1r* gene was used. *p < .05 compared to NPs treatment.

### Efficient cellular uptake of NPs-siRNA into breast cancer cells

Successful internalization of NPs-siRNA by cells with subsequent release of siRNA in cytosol is the most crucial step for effective silencing of target mRNA. As shown in Figure 1(b), the cellular uptake of NPs-siRNA formed with 3 mM of exogenous CaCl_2_ was compared with untreated/NPs-treated and free siRNA-treated MCF-7 cells. The corresponding light photographs (top panel) showed the density of cells in a particular area used to capture fluorescent images (bottom panel). Cells that were kept untreated or treated with NPs or free siRNA did not show any fluorescence under microscope (Figure 1(b), ii, iv, vi) after washing out extracellular particles with EDTA. On the other hand, cells treated with NPs-siRNA showed diffusive pattern of fluorescence (Figure 1(b), viii) after the washing, thus confirming internalization of NPs-siRNA in breast cancer cells, as shown previously in cervical cancer cells (Hossain et al., 2010). The intracellular components from the cells treated with NPs-siRNA also showed significant (*p* = .02) increase in fluorescent intensity compared to those of both untreated and NPs-treated cells (Figure 1(c)). Carbonate apatite NPs are thus capable of carrying electrostatically associated siRNA inside the cells through endocytosis as other non-viral vector and finally, releasing the bound siRNA from the endosome to the cytosol through pH-responsive self-dissolution.

### NPs-GFR siRNA(s) complexes enhance cytotoxicity and apoptosis in breast cancer cells

As GFRs have profound roles in cancer progression, delivery of siRNA(s) would be beneficial to reduce aggressiveness of cancer cells by suppressing growth and inducing cell death. Figure 2 shows the treatment outcomes of the NPs-negative siRNA and NPs-siRNA targeting single *GFR* gene on CV after 2d incubation. Cells showed 80–90% viability when treated with NPs. However, when cells were treated with NPs-bound negative control siRNA, there was no difference observed in the number of viable cells compared to NPs treatment (Figure 2(a)). NPs-bound single siRNA against *egfr1*, *erbb2*, and *igf1r* gene showed 75.73%, 86.36%, and 78.11% (Figure 2(b–d), respectively) viability, respectively, compared to untreated cells. siRNA targeting *egfr1* gene showed significant (*p* = .03) decrease in viability and siRNA targeting *erbb2* gene showed a trend in decreasing viability (statistically not significant) compared to respective NPs treatment, However, no improved outcome was observed for *igf1r*-targeting siRNA.

**Figure 3.**
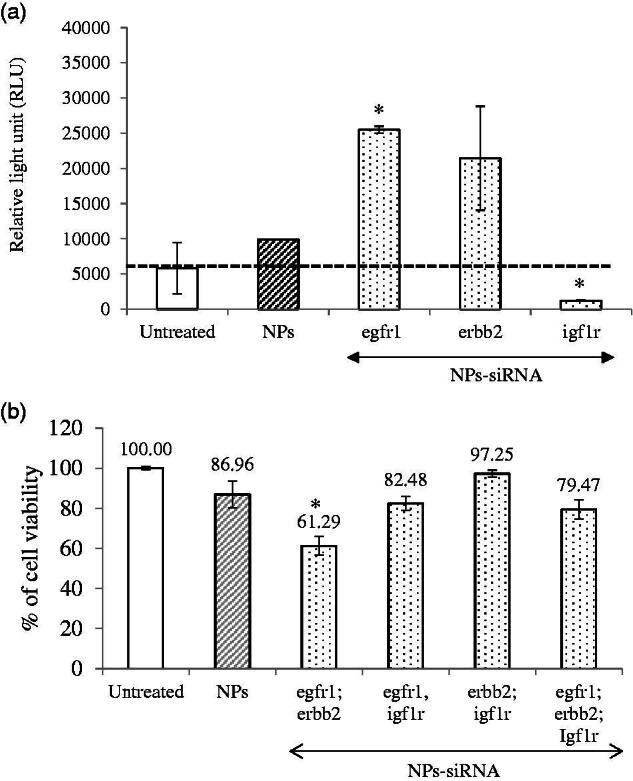
(a) Effect of NPs-loaded siRNA against single gene on caspase-mediated luminescence signal in MCF-7 cells. Cells were treated with media (untreated), NPs and NPs-siRNA (1 nM each siRNA against single *GFR* gene) formed with 3 mM of CaCl_2_. Data were represented as mean ± SD for duplicate samples. (b) Effect of NPs-loaded siRNAs against combination of multiple *GFR* genes (*egfr1, erbb2*, and *igf1r* genes) on viability of MCF-7 cells. Cells were treated with media (untreated), NPs and NPs-siRNAs (1 nM each) formed with 3 mM CaCl_2_. Values are represented as % of cell viability compared to untreated cells for triplicate samples. *p < .05 compared to NPs treatment.

Apoptotic death in the form of caspase-mediated luminescence signal was quantified in the cells treated with NPs complexed with 1 nM of single siRNA against *GFR* genes (Figure 3(a)). The relative light unit (RLU) was increased in the cells treated with NPs-siRNA against *egfr1* (significant, *p* = .02) or *erbb2* (not statistically significant) gene compared to untreated and NPs-treated groups (threshold level was shown by black dot line), whereas the signal was significantly (*p* = .02) reduced below the threshold when the cells were treated with NPs-siRNA against *igf1r* gene, correlating with the cytotoxic effect of the siRNA formulation.

Silencing of *egfr1* gene which is expressed in MCF-7 breast cancer cells (Campiglio et al., 2004) showed higher enhancement in cytotoxicity (Table 2). The effect was solely due to selective silencing of the genes with functionally validated siRNAs, as the NPs-complexed negative control siRNA did not show any toxicity on the cells. EGFR1 and Her2 protect the cells from apoptosis by various mechanisms (Kumar et al., 1996; Rajah et al., 1997; Butt et al., 1999; Dent et al., 1999; Kothari et al., 2003; Reginato et al., 2003) Therefore, silencing of these two *GFR* genes resulted in induction of caspase-7-mediated apoptotic pathway, as the luminescent signals increased after 2 d of the treatment compared to the untreated and NPs-treated cells. MCF-7 cells express low level of EGFR1 protein (Subik et al., 2010), while there is controversy about the expression of ERBB2 on MCF-7 cells. Some studies showed that the expression of Her2 in MCF-7 cells increases under certain conditions (Kumar et al., 1996; Knowlden et al., 2003). In our study, we could detect Her2 expression on MCF-7 cells by Western blot (data not shown). The expression level of Her2 is noticeably low in MCF-7 cells, which could account for the failure of some studies to detect it. Thus, it is fascinating that silencing the poorly expressed *EGFR1* and *ERBB2* genes rendered the cells to be less viable. It is very likely that knocking down an overexpressed gene would be more challenging than a poorly expressed gene. The silencing effect on cytotoxicity was less prominent for *IGF1R* that is expressed highly in MCF-7 cells (Stephen et al., 2001; Chong et al., 2006). Moreover, *igf1r* siRNA significantly decreased caspase-7-initiated signal instead of increasing, further supporting our observation with CV study. It was previously shown that inhibition of IGF1R using specific inhibitor reduced p53-mediated apoptosis by increasing stability of p53 (Xiong et al., 2007). Thus, it might be possible that silencing of this gene decreased p53-mediated apoptosis, thus showing less caspase 7-mediated signal which is up-stream to P53 pathway.

Different combinations of the siRNAs against three *GFR* genes were used to fabricate NPs-siRNAs for testing any potential synergistic effect on cytotoxicity in MCF-7 cells (Figure 3(b)). Some combinations of the siRNAs showed lower CV than untreated and NPs-treated cells, with the most significant and prominent reduction achieved with the siRNAs against *egfr1* and *erbb2* genes (*p* = .01) compared to NPs-treated cells, conferring 25.67% net cytotoxicity (Figure 3(b); Table 2). As stated earlier, EGFR1 forms homodimer and heterodimer with ERBB2 upon activation by ligand interaction (Ullrich et al., 1984; Yamamoto et al., 1986). ERBB2 cannot form homodimer and needs EGFR1 to form heterodimer in order to be activated (Yarden, 2001). Since, the heterodimer shows the strongest biological activity (Yarden, 2001), silencing of both receptors had more toxic effects than their single counter parts.

NPs-siRNAs against *egfr1* and *igf1r* showed similar viability as NPs treatment (Figure 3(b)). Our finding is partly contradicting with the finding of Kaulfuss et al. (2009), who showed that silencing of both *egfr1* and *igf1r* expression in colorectal cancer cells induced apoptosis by Caspase-3, and -7 pathways. However, the consequence of silencing of a particular gene depends on cell types, the expression level of GFRs, the vehicle system used for delivery of siRNA(s) and knockdown efficacy of the siRNA(s). NPs-siRNAs against *erbb2* and *igf1r* genes also failed to show any further cytotoxicity. Interestingly, when siRNAs against all three *GFR* genes were used the enhancement of cytotoxicity was 7.49% which was not as prominent as that achieved with *egfr1* and *erbb2* siRNAs. These results suggest that siRNA against *igf1r* gene might inhibit apoptotic signals, thus annulling the concerted effects of the siRNAs against other two genes.

### NPs-GFR siRNA(s) complexes regress tumor outgrowth in mouse model

**Figure 4.**
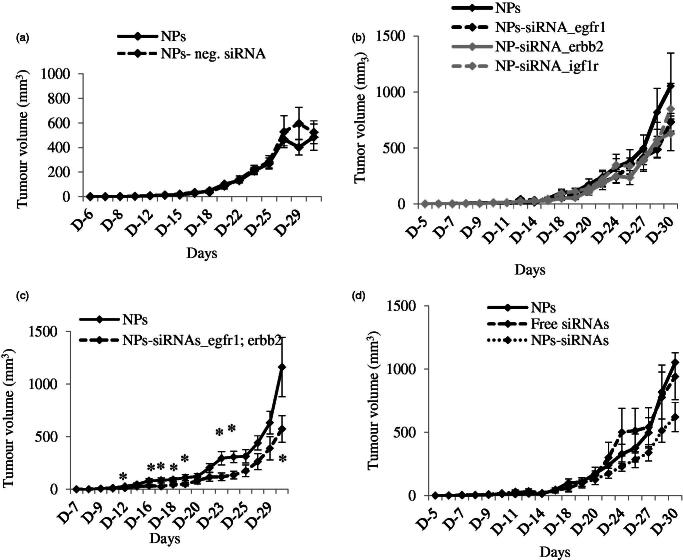
(a) Effect of NPs-negative siRNA on tumor outgrowth in 4T1-cells induced mouse model. Mice were treated intravenously through tail-vein injection with 100 µL of either NPs or NPs-negative siRNA (50 nM) formed with 70 mM of CaCl_2_. (b–d) Effect of NPs-siRNA(s) against single or multiple *GFR* genes on tumor outgrowth in 4T1-cells induced mouse model designed. Mice were treated intravenously through tail-vein injection with 100 µL of free/NPs-siRNA(s) formed in 70 mM of CaCl_2_ and 50 nM of individual siRNA(s). Tumor outgrowth of mice treated with panel (b), NPs-siRNA against single *GFR* gene, (c) NPs-siRNAs against *egfr1* and *erbb2* genes, and (d) free/NP-siRNAs against all three *GFR* genes. Six mice/group were used and data were represented as mean ± SD. *p < .05 compared to NPs treatment.

A syngeneic mouse model of 4T1-cells-induced breast tumor was used to investigate the effects of NPs-siRNAs against *GFR* genes on tumor regression. Mice treated with NPs-negative siRNA did not show any significant difference in body weights (Supplementary Figure 4aFigure 4.) and tumor outgrowth compared with control (NPs-treated) mice (Figure 4(a)).

Next, mice were treated with NPs-siRNA (50 nM each) against single *GFR* gene, *egfr1*, *erbb2* or *igf1r* and the tumor volume was observed (Figure 4(b)). The trend of tumor outgrowths with time for all of the three single siRNAs in NPs-bound forms was lower than the NPs alone, although the difference was not statistically significant. As NPs-siRNAs against *egfr1* and *erbb2* genes showed the highest enhancement in cytotoxicity, mice were treated with this combination and compared with NPs treated mice (Figure 4(c)). As shown, this arrangement of NPs-siRNAs against *egfr1* and *erbb2* significantly reduced the tumor with time compared to control after first dose till end of the study, more specifically (shown by asterisks) at day 12, 16, 17, 18, 19, 23, 24, and 30 with *p* values of 0.038, 0.036, 0.025, 0.037, 0.047, 0.018, 0.012, and 0.041 respectively. siRNAs against *egfr1* or *erbb2* gene showed increase in Caspase-mediated luminescence signal, cytotoxicity and significant regression in tumor volume, thus shedding light on the potential roles of the siRNAs in treating breast cancer. Successively, mice were also treated with free/NPs-siRNAs against all three *GFR* genes to check the effects on tumor reduction (Figure 4(d)). The tumor outgrowth over time for NPs-siRNAs-treated group was smaller than the NPs- and free siRNAs-treated group, although the difference was not so significant in the beginning of the study. However, there was a significant reduction in tumor growth at the end as a result of the combined knockdown effect of all three receptors, compared to the control groups. Free GFR siRNAs also did not show effect in reducing tumor size as free siRNAs could be degraded by the serum nucleases and subjected to renal clearance before reaching to its target site (Layzer et al., 2004; Soutschek et al., 2004; Morrissey et al., 2005; Gao et al., 2009). When multiple siRNAs are delivered with NPs, siRNA that silences a gene not crucial for tumor regression might compete for and saturate the siRNA processing machinery within cells (Hutvagner et al., 2004; Grimm et al., 2006; Koller et al., 2006; Castanotto et al., 2007; Vickers et al., 2007; Khan et al., 2009), thus leading to lesser effects of the therapeutically important siRNA(s). The body weights for all these groups remain unchanged (Supplementary Figure 4 b–d) which confirms that NPs-siRNA(s) treatment does not have an influence on gross body weight.

In a nutshell, silencing of *egfr1* and *erbb2* genes by the specific siRNAs delivered by carbonate apatite NPs reduce CV induces apoptotic cell death and decrease the tumor burden in mice, thus suggesting that NPs-loaded siRNAs targeting *egfr1* and *erbb2* genes could be a potential molecular therapeutic to treat breast cancer.

## Supplementary Material

IDRD_Chowdhury_et_al_Supplemental_Content.docx

## References

[CIT0001] Allen LF, Lenehan PF, Eiseman IA, et al. (2002). Potential benefits of the irreversible pan-erbB inhibitor, CI-1033, in the treatment of breast cancer. Semin Oncol 29:11–21. 10.1053/sonc.2002.34049 12138393

[CIT0002] Butt AJ, Firth SM, Baxter RC. (1999). The IGF axis and programmed cell death. Immunol Cell Biol 77:256–62. 10361258 10.1046/j.1440-1711.1999.00822.x

[CIT0003] Campiglio M, Locatelli A, Olgiati C, et al. (2004). Inhibition of proliferation and induction of apoptosis in breast cancer cells by the epidermal growth factor receptor (EGFR) tyrosine kinase inhibitor ZD1839 (‘Iressa’) is independent of EGFR expression level. J Cell Physiol 198:259–68. 14603528 10.1002/jcp.10411

[CIT0004] Castanotto D, Sakurai K, Lingeman R, et al. (2007). Combinatorial delivery of small interfering RNAs reduces RNAi efficacy by selective incorporation into RISC. Nucleic Acids Res 35:5154–64. 17660190 10.1093/nar/gkm543 PMC1976469

[CIT0005] Chong YM, Colston K, Jiang WG, et al. (2006). The relationship between the insulin-like growth factor-1 system and the oestrogen metabolising enzymes in breast cancer tissue and its adjacent non-cancerous tissue. Breast Cancer Res Treat 99:275–88. 16752221 10.1007/s10549-006-9215-y

[CIT0006] Chua MJ, Tiash S, Fatemian T, et al. (2013). Carbonate apatite-facilitated intracellular delivery of c-ROS1 small interfering RNA sensitises MCF-7 breast cancer cells to cisplatin and paclitaxel. OA Cancer 1:1–9.

[CIT0007] Dent P, Reardon DB, Park JS, et al. (1999). Radiation-induced release of transforming growth factor alpha activates the epidermal growth factor receptor and mitogen-activated protein kinase pathway in carcinoma cells, leading to increased proliferation and protection from radiation-induced cell death. Mol Biol Cell 10:2493–506. 10436007 10.1091/mbc.10.8.2493 PMC25480

[CIT0008] Di Fiore PP, Pierce JH, Kraus MH, et al. (1987). erbB-2 is a potent oncogene when overexpressed in NIH/3T3 cells. Science 237:178–82. 2885917 10.1126/science.2885917

[CIT0009] Gao S, Dagnaes-Hansen F, Nielsen EJ, et al. (2009). The effect of chemical modification and nanoparticle formulation on stability and biodistribution of siRNA in mice. Mol Ther 17:1225–33. 19401674 10.1038/mt.2009.91 PMC2835214

[CIT0010] Gee JM, Robertson JF, Gutteridge E, et al. (2005). Epidermal growth factor receptor/HER2/insulin-like growth factor receptor signalling and oestrogen receptor activity in clinical breast cancer. Endocr Relat Cancer 12:S99–S111. 16113104 10.1677/erc.1.01005

[CIT0011] Grimm D, Streetz KL, Jopling CL, et al. (2006). Fatality in mice due to oversaturation of cellular microRNA/short hairpin RNA pathways. Nature 441:537–41. 16724069 10.1038/nature04791

[CIT0012] Guy CT, Cardiff RD, Muller WJ. (1996). Activated neu induces rapid tumor progression. J Biol Chem 271:7673–8. 8631805 10.1074/jbc.271.13.7673

[CIT0013] Guy CT, Webster MA, Schaller M, et al. (1992). Expression of the neu protooncogene in the mammary epithelium of transgenic mice induces metastatic disease. Proc Natl Acad Sci USA 89:10578–82. 1359541 10.1073/pnas.89.22.10578 PMC50384

[CIT0014] Herbst RS, Shin DM. (2002). Monoclonal antibodies to target epidermal growth factor receptor-positive tumors: a new paradigm for cancer therapy. Cancer 94:1593–611. 11920518 10.1002/cncr.10372

[CIT0015] Hossain S, Stanislaus A, Chua MJ, et al. (2010). Carbonate apatite-facilitated intracellularly delivered siRNA for efficient knockdown of functional genes. J Control Release 147:101–8. 20620182 10.1016/j.jconrel.2010.06.024

[CIT0016] Hurtado A, Holmes KA, Geistlinger TR, et al. (2008). Regulation of ERBB2 by oestrogen receptor-PAX2 determines response to tamoxifen. Nature 456:663–6. 19005469 10.1038/nature07483 PMC2920208

[CIT0017] Hutvagner G, Simard MJ, Mello CC, Zamore PD. (2004). Sequence-specific inhibition of small RNA function. PLoS Biol 2:E98 15024405 10.1371/journal.pbio.0020098 PMC350664

[CIT0018] Kaulfuss S, Burfeind P, Gaedcke J, Scharf JG. (2009). Dual silencing of insulin-like growth factor-I receptor and epidermal growth factor receptor in colorectal cancer cells is associated with decreased proliferation and enhanced apoptosis. Mol Cancer Ther 8:821–33. 19372555 10.1158/1535-7163.MCT-09-0058

[CIT0019] Khan AA, Betel D, Miller ML, et al. (2009). Transfection of small RNAs globally perturbs gene regulation by endogenous microRNAs. Nat Biotechnol 27:549–55. 19465925 10.1038/nbt.1543 PMC2782465

[CIT0020] Klijn JG, Berns PM, Schmitz PI, Foekens JA. (1992). The clinical significance of epidermal growth factor receptor (EGF-R) in human breast cancer: a review on 5232 patients. Endocr Rev 13:3–17. 1313356 10.1210/edrv-13-1-3

[CIT0021] Knowlden JM, Hutcheson IR, Jones HE, et al. (2003). Elevated levels of epidermal growth factor receptor/c-erbB2 heterodimers mediate an autocrine growth regulatory pathway in tamoxifen-resistant MCF-7 cells. Endocrinology 144:1032–44. 12586780 10.1210/en.2002-220620

[CIT0022] Koller E, Propp S, Murray H, et al. (2006). Competition for RISC binding predicts in vitro potency of siRNA. Nucleic Acids Res 34:4467–76. 16945958 10.1093/nar/gkl589 PMC1636362

[CIT0023] Kothari S, Cizeau J, McMillan-Ward E, et al. (2003). BNIP3 plays a role in hypoxic cell death in human epithelial cells that is inhibited by growth factors EGF and IGF. Oncogene 22:4734–44. 12879018 10.1038/sj.onc.1206666

[CIT0024] Kumar R, Mandal M, Lipton A, et al. (1996). Overexpression of HER2 modulates bcl-2, bcl-XL, and tamoxifen-induced apoptosis in human MCF-7 breast cancer cells. Clin Cancer Res 2:1215–19. 9816290

[CIT0025] Kunnath AP, Kamaruzman NI, Chowdhury EH. (2014a). Nanoparticle-facilitated intratumoral delivery of Bcl-2/IGF-1R siRNAs and p53 gene synergistically inhibits tumor growth in immunocompetent mice. J Nanomed Nanotechnol S9:001.

[CIT0026] Kunnath AP, Tiash S, Fatemian T, et al. (2014b). Intracellular delivery of ERBB2 siRNA and p53 gene synergistically inhibits the growth of established tumor in an immunocompetent mouse. J Cancer Sci Ther 6:99–104.

[CIT0027] Layzer JM, McCaffrey AP, Tanner AK, et al. (2004). In vivo activity of nuclease-resistant siRNAs. RNA 10:766–71. 15100431 10.1261/rna.5239604 PMC1370566

[CIT0028] Morrissey DV, Lockridge JA, Shaw L, et al. (2005). Potent and persistent in vivo anti-HBV activity of chemically modified siRNAs. Nat Biotechnol 23:1002–7. 16041363 10.1038/nbt1122

[CIT0029] Nahta R, Hortobagyi GN, Esteva FJ. (2003). Growth factor receptors in breast cancer: potential for therapeutic intervention. Oncologist 8:5–17. 10.1634/theoncologist.8-1-5 12604728

[CIT0030] Nahta R, Yuan LX, Zhang B, et al. (2005). Insulin-like growth factor-I receptor/human epidermal growth factor receptor 2 heterodimerization contributes to trastuzumab resistance of breast cancer cells. Cancer Res 65:11118–28. 16322262 10.1158/0008-5472.CAN-04-3841

[CIT0031] Rajah R, Valentinis B, Cohen P. (1997). Insulin-like growth factor (IGF)-binding protein-3 induces apoptosis and mediates the effects of transforming growth factor-beta1 on programmed cell death through a p53- and IGF-independent mechanism. J Biol Chem 272:12181–8. 9115291 10.1074/jbc.272.18.12181

[CIT0032] Reginato MJ, Mills KR, Paulus JK, et al. (2003). Integrins and EGFR coordinately regulate the pro-apoptotic protein Bim to prevent anoikis. Nat Cell Biol 5:733–40. 12844146 10.1038/ncb1026

[CIT0033] Slamon DJ, Clark GM, Wong SG, et al. (1987). Human breast cancer: correlation of relapse and survival with amplification of the HER-2/neu oncogene. Science 235:177–82. 3798106 10.1126/science.3798106

[CIT0034] Soutschek J, Akinc A, Bramlage B, et al. (2004). Therapeutic silencing of an endogenous gene by systemic administration of modified siRNAs. Nature 432:173–8. 15538359 10.1038/nature03121

[CIT0035] Stephen RL, Shaw LE, Larsen C, et al. (2001). Insulin-like growth factor receptor levels are regulated by cell density and by long term estrogen deprivation in MCF7 human breast cancer cells. J Biol Chem 276:40080–6. 11457860 10.1074/jbc.M105892200

[CIT0036] Subik K, Lee JF, Baxter L, et al. (2010). The expression patterns of ER, PR, HER2, CK5/6, EGFR, Ki-67 and AR by immunohistochemical analysis in breast cancer cell lines. Breast Cancer (Auckl) 4:35–41. 20697531 PMC2914277

[CIT0037] Tiash S, Othman I, Rosli R, Chowdhury EH. (2014). Methotrexate- and cyclophosphamide-embedded pure and strontiumsubstituted carbonate apatite nanoparticles for augmentation of chemotherapeutic activities in breast cancer cells. Curr Drug Deliv 11:214–22. 24328684 10.2174/1567201810666131211101819

[CIT0038] Ullrich A, Coussens L, Hayflick JS, et al. (1984). Human epidermal growth factor receptor cDNA sequence and aberrant expression of the amplified gene in A431 epidermoid carcinoma cells. Nature 309:418–25. 6328312 10.1038/309418a0

[CIT0039] Vickers TA, Lima WF, Nichols JG, Crooke ST. (2007). Reduced levels of Ago2 expression result in increased siRNA competition in mammalian cells. Nucleic Acids Res 35:6598–610. 17905815 10.1093/nar/gkm663 PMC2095815

[CIT0040] Voldborg BR, Damstrup L, Spang-Thomsen M, Poulsen HS. (1997). Epidermal growth factor receptor (EGFR) and EGFR mutations, function and possible role in clinical trials. Ann Oncol 8:1197–206. 9496384 10.1023/a:1008209720526

[CIT0041] Waterfield MD. (1989). Growth factor receptors. Br Med Bull 45:541–53. 2557119 10.1093/oxfordjournals.bmb.a072341

[CIT0042] Xiong L, Kou F, Yang Y, Wu J. (2007). A novel role for IGF-1R in p53 mediated apoptosis through translational modulation of the p53-Mdm2 feedback loop. J Cell Biol 178:995–1007. 17846171 10.1083/jcb.200703044 PMC2064623

[CIT0043] Yamamoto T, Ikawa S, Akiyama T, et al. (1986). Similarity of protein encoded by the human c-erb-B-2 gene to epidermal growth factor receptor. Nature 319:230–4. 3003577 10.1038/319230a0

[CIT0044] Yarden Y. (2001). Biology of HER2 and its importance in breast cancer. Oncology 61:1–13. 10.1159/000055396 11694782

[CIT0045] Yarden Y. (2001). The EGFR family and its ligands in human cancer. signalling mechanisms and therapeutic opportunities. Eur J Cancer 37:S3–S8. 10.1016/s0959-8049(01)00230-1 11597398

[CIT0046] Yee D. (2002). The insulin-like growth factor system as a treatment target in breast cancer. Semin Oncol 29:86–95. 12138402 10.1053/sonc.2002.34060

[CIT0047] Yonesaka K, Zejnullahu K, Okamoto I, et al. (2011). Activation of ERBB2 signaling causes resistance to the EGFR-directed therapeutic antibody cetuximab. Sci Transl Med 3:99ra86 10.1126/scitranslmed.3002442 PMC3268675 21900593

