# Supplementary figures and images for: Carbonate apatite nanoparticles carry siRNA(s) targeting growth factor receptor genes egfr1 and erbb2 to regress mouse breast tumor
Source: Drug Deliv. 2017 Nov 9;24(1):1721–30. doi: 10.1080/10717544.2017.1396385 (PMC8240997; doi:10.1080/10717544.2017.1396385)

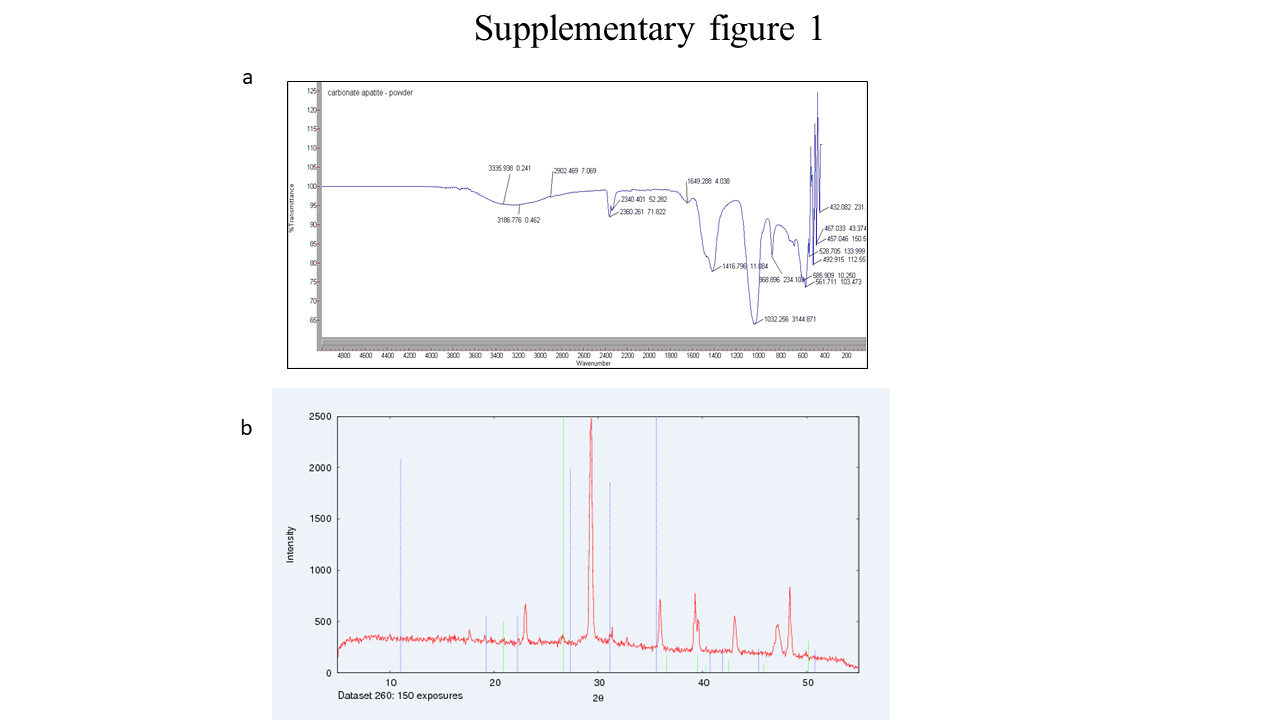


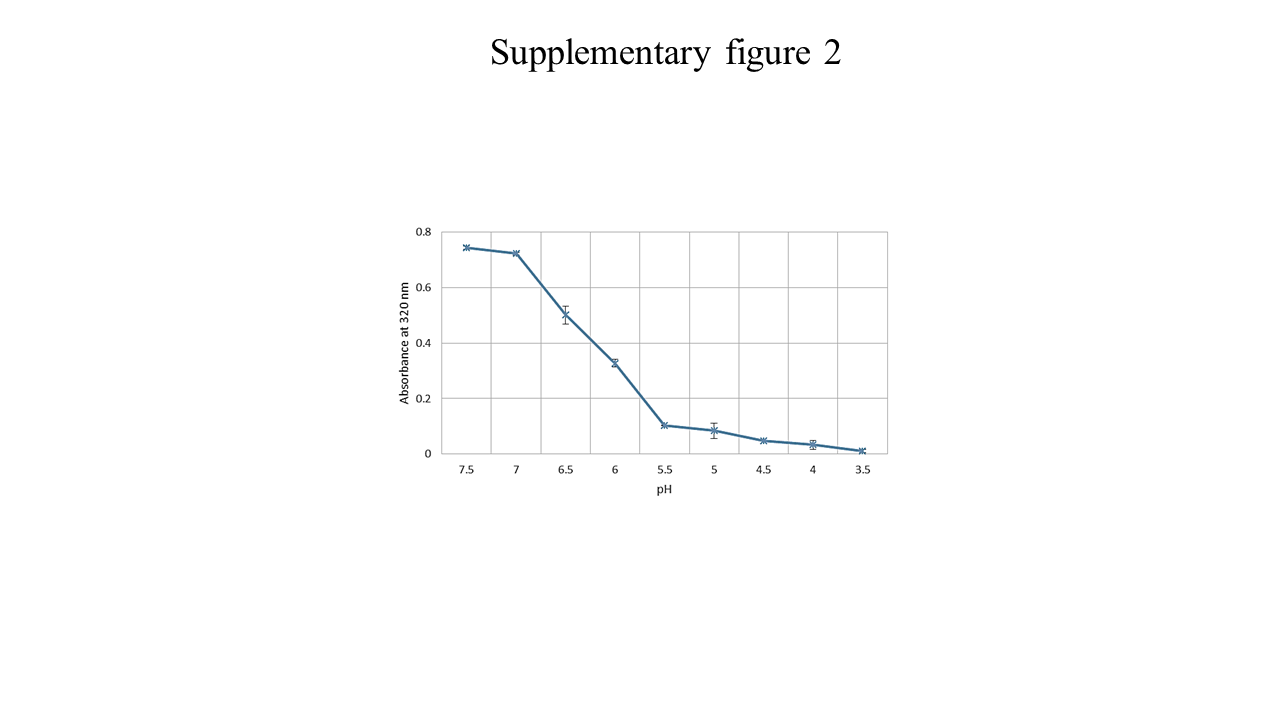


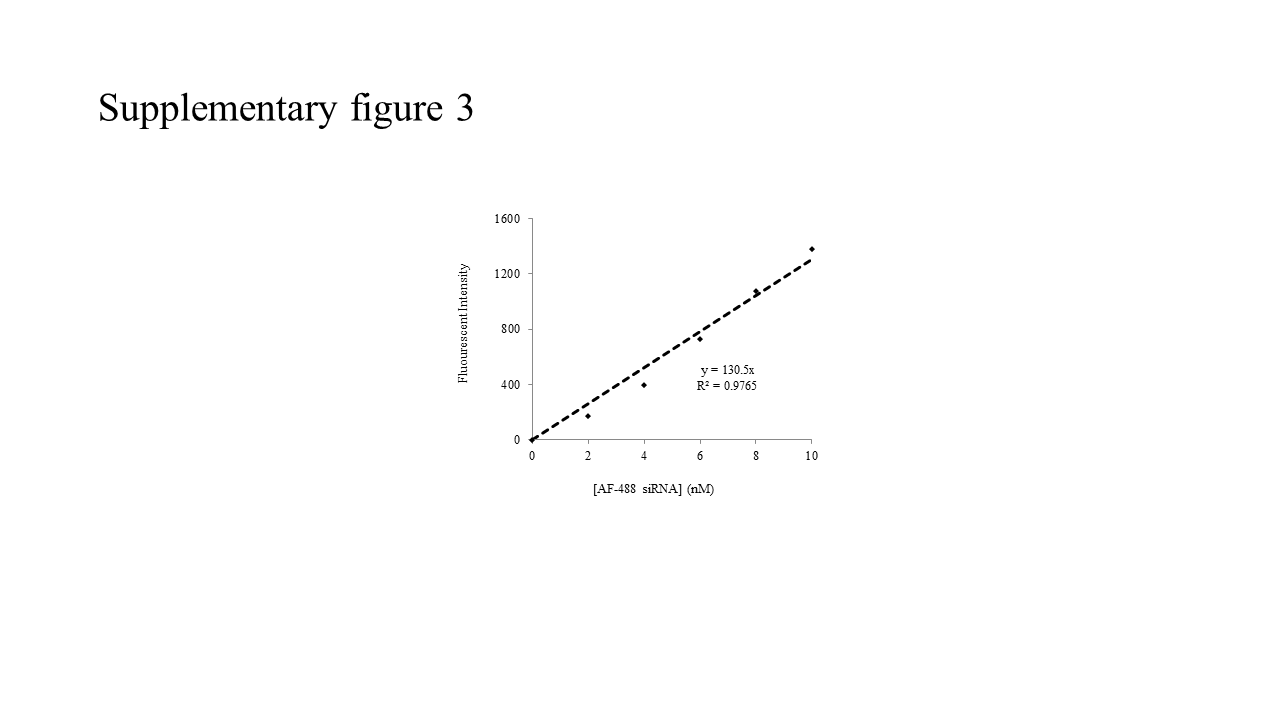


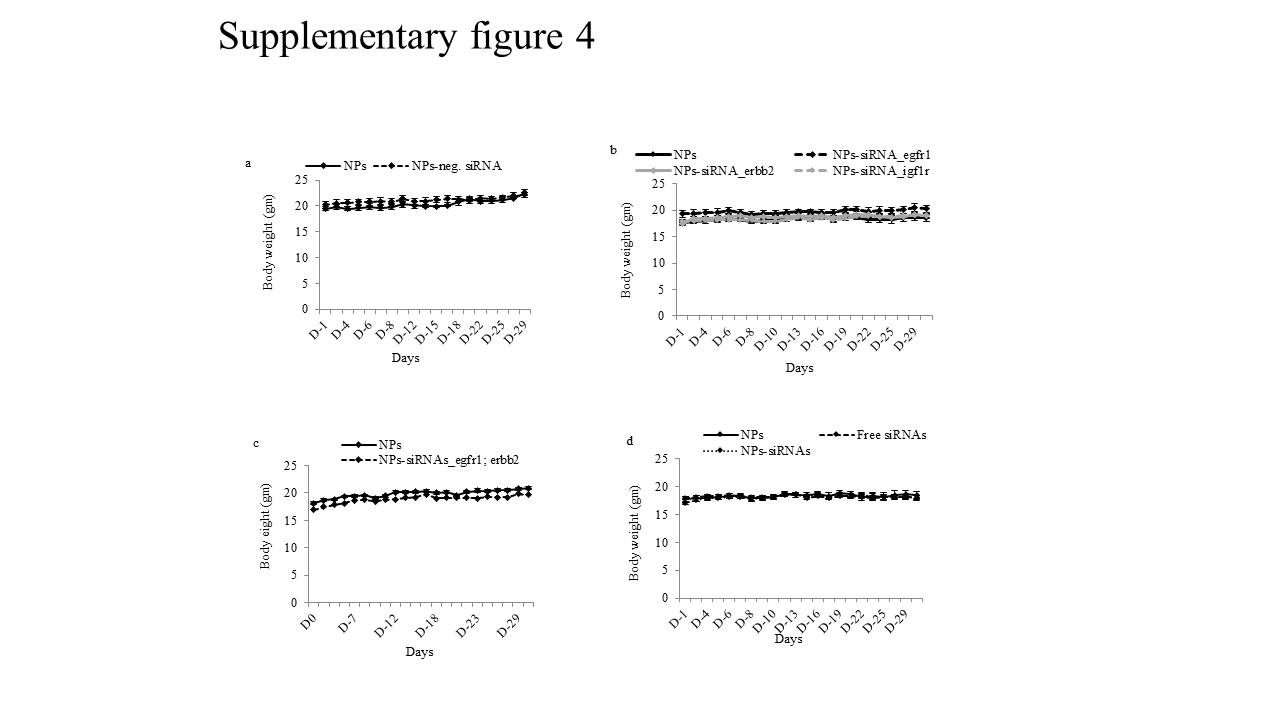

Supplement: IDRD_Chowdhury_et_al_Supplemental_Content.docx [file IDRD_A_1396385_SM9688.docx]
